# Off-Label Treatment of Alopecia Areata—Retrospective Study

**DOI:** 10.3390/biomedicines14020367

**Published:** 2026-02-05

**Authors:** Julia Sternicka-Rohde, Leszek Bieniaszewski, Natalia Krzyżaniak, Roman J. Nowicki, Dorota Purzycka-Bohdan

**Affiliations:** 1 Department of Dermatology, Venereology and Allergology, Medical University of Gdansk, 80-210 Gdansk, Poland; 2 Department of Dermatology, Venereology and Allergology, University Clinical Centre, Smoluchowskiego Street 17, 80-214 Gdansk, Poland; 3 Clinical Physiology Unit, Medical Simulation Centre, Medical University of Gdansk, Dębowa Street 25, 80-204 Gdansk, Poland

**Keywords:** alopecia areata, off-label, treatment, baricitinib, ritlecitinib, dermatology

## Abstract

**Background/Objectives**: Alopecia areata is an autoimmune disorder affecting approximately 2% of the global population and is associated with a substantial impairment in quality of life. Owing to the limited number of approved therapeutic options, off-label pharmacotherapy is frequently employed in clinical practice when managing this disease. **Methods**: This retrospective study analyzed electronic medical records of patients treated for alopecia areata at the University Clinical Centre in Gdańsk between 2014 and 2024 to characterize the epidemiological profile and real-world treatment patterns. **Results**: A total of 334 affected patients were identified, including 199 diagnosed exclusively with alopecia areata and others presenting with immune-mediated comorbidities, most commonly atopic dermatitis and psoriasis. Among patients with isolated disease, women were more frequently affected and were older at diagnosis than men. Most individuals were managed in the outpatient setting, and demographic characteristics remained stable throughout the study period. Off-label pharmacotherapy was used in 77.9% of all patients and in 99.4% of those receiving drug treatment, with no significant associations observed between off-label use and age, sex, place of residence, or calendar year. Glucocorticosteroids, administered both topically and systemically, were the most commonly prescribed off-label agents (65.3%), and monotherapy was the predominant treatment strategy. **Conclusions**: These findings highlight the extensive reliance on off-label therapies in routine management of alopecia areata in a real-world European clinical setting.

## 1. Introduction

Alopecia areata (AA) is an autoimmune disease belonging to the non-scarring alopecia group [1]. Despite its low prevalence, estimated to be 2% in the global population, it poses a significant challenge for clinicians due to its consequential impact on patients’ quality of life [2,3]. The pathogenesis of alopecia areata (AA) remains incompletely understood; however, current evidence suggests a multifactorial etiology involving genetic susceptibility, autoimmune mechanisms, dysregulation of immune responses, and heightened inflammatory activity [4,5]. The clinical picture is most often characterized by patches of hair loss, but may also manifest as a lack of part of the hair along the sides and back of the scalp in a pattern resembling a snake or a band (alopecia ophiasis), loss of all scalp hair (alopecia totalis), or loss of all scalp and body hair (alopecia universalis) [6]. Trichoscopic examination of alopecia areata reveals characteristic features such as exclamation mark hairs, dystrophic hairs, and yellow dots [6]. Disease severity is assessed using the Severity of Alopecia Tool (SALT) and Alopecia Areata Scale (AAS) [1]. A SALT score of 20 or higher, as well as moderate to severe forms of alopecia areata according to the AAS, constitute an indication for systemic treatment [1]. Currently, the range of medications used in the therapy of AA includes only two drugs approved by the European Medicines Agency (EMA) and the Food and Drug Administration (FDA). These are Janus kinase inhibitors: baricitinib (JAK 1/2 inhibitor) and ritlecitinib (JAK 3/TEC inhibitor) [1]. According to the Summary of Product Characteristics (SmPC), the former should be used in adults, while the latter can be used in patients aged 12 years and older, in both cases in those with severe AA [7,8]. These medications have been on the market for a relatively short time and, moreover, they do not meet the therapeutic needs of all patients. Therefore, when managing AA, physicians often resort to off-label medications.

Off-label treatment is the use of a medicine that goes beyond the SmPC guidelines [9,10,11,12,13,14,15,16,17]. This may refer to the indication, age group, dose, form, or route of administration [9,12,13]. Additional conditions that must be met to constitute off-label therapy are that the drug in question is registered in a given country and that scientific evidence exists to support the use of the drug in a specific disease entity (guidelines, high-quality scientific papers) [9,10,14]. This distinguishes the discussed form of therapy from unlicensed use of drugs and medical experiments [9,10,14]. Furthermore, this form of treatment cannot be performed without the patient’s informed consent, which must be obtained before the therapy is implemented [9]. Off-label therapy is most often considered when standard treatments approved within the SmPC are either unavailable or fail to meet the patient’s clinical needs [11]. This is particularly relevant in the management of AA, for which approved therapeutic options remain limited [1]. As a result, off-label medications are frequently used in clinical practice. The present study aims to evaluate the prevalence of off-label treatment in patients with AA in Northern Poland and to assess clinical correlations with this form of therapy, based on real-world data collected over 10 years at the Department of Dermatology, Venereology and Allergology and the Dermatology Outpatient Clinic in Gdańsk.

## 2. Materials and Methods

A retrospective study was carried out utilizing electronic medical records (EMR) of patients diagnosed with AA, who received care at either the Department of Dermatology, Venereology and Allergology or the Dermatology Outpatient Clinic in Gdańsk in the years 2014–2024. The extracted data included sociodemographic information—sex, age and place of residence—details on comorbidities, administered therapies, discharge summaries, medical recommendations, prescribed medications, physicians’ notes, and visit records. This information covered the entire treatment period for each patient within the aforementioned units. Patient age was recorded as the age at first observation at either the inpatient or outpatient dermatology units. Similarly, the place of residence, categorized as urban or rural, was determined based on the location provided during the patient’s initial contact with the Department or Clinic. To classify the medications used, trade names identified in the records were cross-referenced with the Summary of Product Characteristics (SmPC) documents. Based on this, medications were grouped to their international nonproprietary names and classified accordingly. Drugs were further categorized as either approved (on-label) or unapproved (off-label) for use in the treatment of AA (Table 1). Only indication-related discrepancies with the SmPC were considered when classifying prescriptions as off-label.

To ensure clarity in interpreting the treatment approaches, individuals with coexisting conditions were excluded from the statistical analysis of implemented therapy. Patients were subsequently divided into four distinct groups: those who received no pharmacological treatment, those treated exclusively with drugs approved for AA (on-label), those who received a combination of on-label and off-label therapy and those treated solely with off-label medications. An analysis was then conducted to determine the frequency of use of the medication groups outlined in Table 1, with a focus on the distinction between on-label and off-label usage. The frequency analysis was performed at the therapy level rather than the patient level. Because multiple therapies could be administered concurrently to a single patient, the denominator for the calculations was the total number of therapies used. As a result, the sum of percentages exceeds 100%. Statistical analysis also examined associations between the frequency of off-label prescribing and patient characteristics, including sex, age, place of residence, and type of healthcare contact (inpatient or outpatient). The chi-square (Χ^2^) test was applied to assess potential correlations between qualitative variables. Clinical outcome metrics such as the SALT score, AAS, and Dermatology Life Quality Index (DLQI) were unavailable in analyzed documentation, so they were not evaluated. To determine whether the frequency of off-label treatment changed over time during the 10-year study period, the Cochran–Mantel–Haenszel (CMH) test was utilized. All statistical procedures were conducted using SAS 9.4 (Enterprise Edition) developed by SAS Institute Inc., Cary, NC, USA. The study protocol followed the ethical standards outlined in the Declaration of Helsinki (1964) and its subsequent amendments and received formal approval from the Independent Bioethics Committee for Scientific Research at the Medical University of Gdańsk (approval number KB/331/2024, issued on 9 July 2024).

## 3. Results

**Figure 1:**
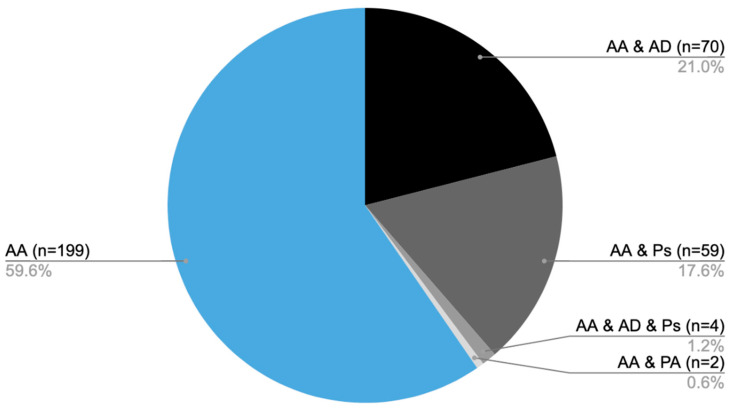
Distribution of patients diagnosed with AA and coexisting conditions: number of patients with AA only (AA), AA with AD (AA&AD), AA with psoriasis (AA&Ps), AA with both atopic dermatitis and psoriasis (AA&AD&Ps), and AA with psoriatic arthritis (AA&PA).

From 2014 to 2024, medical care was provided to 334 patients diagnosed with AA at the Dermatology, Venereology, and Allergology Department, as well as the Dermatology Outpatient Clinic in Gdansk. Of the total, 199 patients were diagnosed solely with AA [AA], while 70 individuals also had atopic dermatitis (AD) [AA&AD], 59 were additionally diagnosed with psoriasis [AA&Ps], and 4 patients presented with all three conditions [AA&AD&Ps]. Furthermore, two individuals had both AA and psoriatic arthritis [AA&PA] (Figure 1).

**Figure 2:**
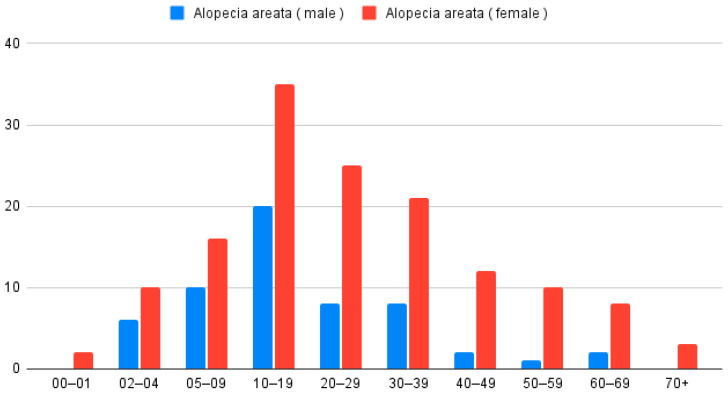
Distribution of patients diagnosed with AA only (*n* = 199), categorized by age group and sex.

In the analysis of patients with only AA (*n* = 199), 142 were women and 57 were men. According to the Z-test for proportion, women were more often diagnosed with AA (*p* < 0.001). The average age for the studied group was 24.8 years. Women diagnosed with AA were older than men (Wilcoxon Two-Sample Test; *p* = 0.01); the mean age of women was 27 years and of men 19.2 years (Figure 2).

When analyzing place of residence, 145 patients (72.9%) lived in urban areas and 54 (27.1%) in rural areas, with a higher proportion coming from cities (Z-test for proportion *p* < 0.001). The age distribution for females and males was similar (Chi-square test *p* = 0.3855). The maximum was observed at 2 and 3 decades of life.

Outpatient care was provided to the majority of patients, accounting for 181 individuals (91%), while 18 patients (9%) received inpatient treatment. A similar percentage of women was observed among outpatients vs. hospitalized patients (Fisher exact test *p* = 0.287).

**Figure 3:**
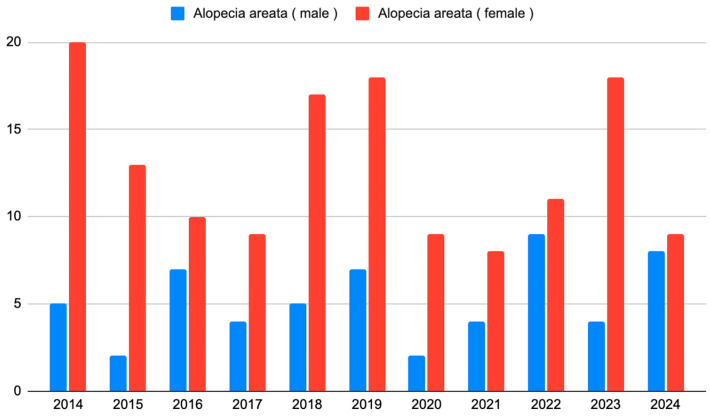
The number of patients with AA who initiated observation each year from 2014 to 2024.

The number of new observations and the gender structure were stable over the years studied (*p* = 0.142, Cochran–Mantel–Haenszel test) (Figure 3). 

**Figure 4:**
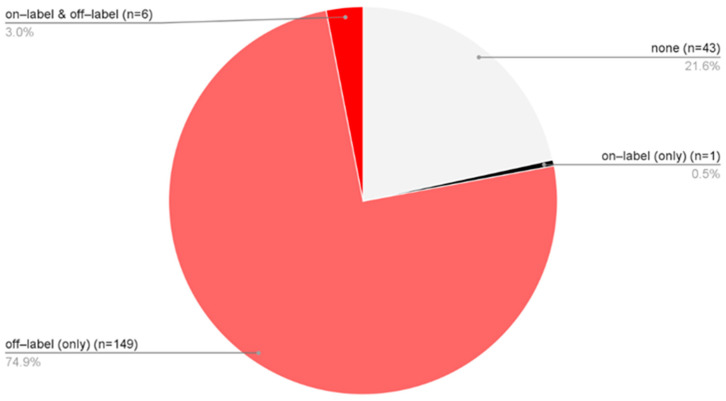
The proportion of only AA patients (*n* = 199) distributed across four treatment categories based on the type of medication used: no pharmacological therapy [none], solely off-label treatment [off-label (only)], a combination of on-label and off-label therapies [on-label & off-label], treatment exclusively in accordance with approved SmPC indications [on-label (only)].

Treatment approaches varied among patients diagnosed solely with AA, allowing classification into four groups: 43 patients received no pharmacological therapy [none], 149 were treated exclusively with off-label medications [off-label (only)], 6 received a combination of on-label and off-label treatments [on-label & off-label], and 1 patient was managed solely in accordance with the SmPCs [on-label (only)] (Figure 4). In total, 77.9% of AA patients underwent some form of off-label therapy. When considering only the subgroup of patients who received pharmacological treatment, 99.4% were managed with off-label therapies.

Among patients treated solely with off-label medications or with combined off-label and on-label therapy, there were 112 women, and 43 were men. Men were treated similarly to women (Fisher exact test, *p* = 0.803). Place of residence was also not associated with a different treatment regimen (Fisher exact test, *p* = 0.418). There was no relationship between off-label treatment and age (*p* = 0.749, Kruskal–Wallis Test).

**Figure 5:**
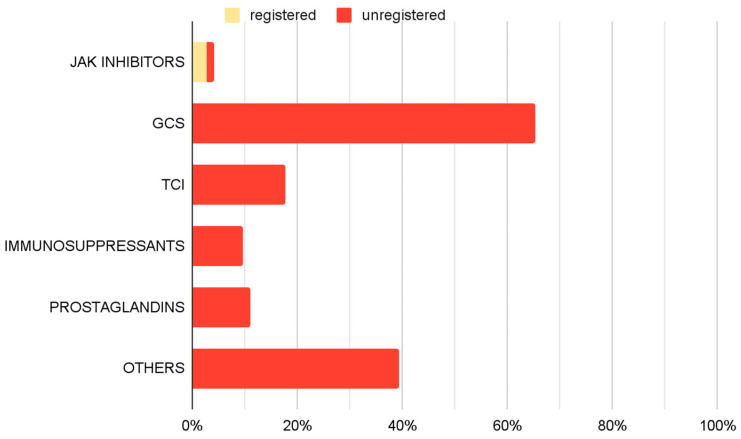
Prevalence of use of specific drug groups among therapies used in treating AA only—therapy-based analyses.

An analysis of pharmacological management revealed a variety of therapeutic approaches (Figure 5). GCS, including both oral and topical formulations, were the most frequently utilized off-label medications, administered in 65.3% of cases. TCIs were part of 17.6% of implemented therapies. Immunosuppressive agents (9.7%) and prostaglandin analogues (11.1%) were also employed. JAK inhibitors were prescribed in accordance with approved SmPC guidelines in 2.8% of cases, and 1.4% outside their approved use. Additionally other off-label treatments were implemented in 39.4% of cases. 

The “Others” treatment category included mesalazine, minoxidil, contact immunotherapies, hydroxychloroquine, and chloroquine. Among the 85 patients treated with agents from this group, minoxidil was administered to 84 patients. In 78 patients (91.8%), minoxidil was the only treatment used within this category.

**Figure 6:**
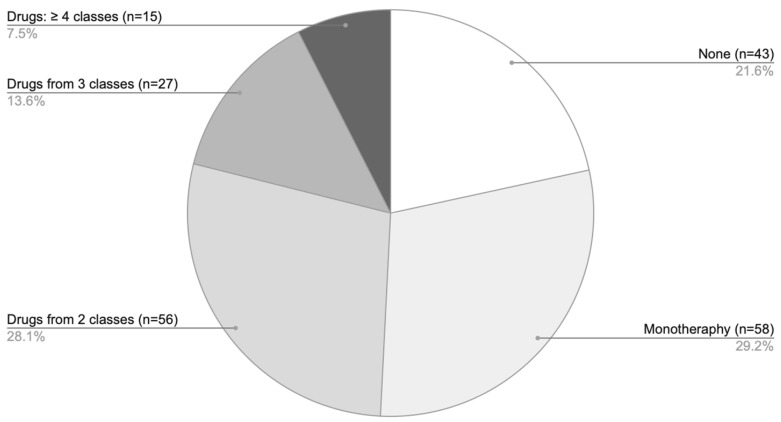
Distribution of pharmacotherapy according to the number of agents used among patients with AA only.

Analysis of pharmacotherapy by the number of agents showed that monotherapy was the predominant treatment approach (Figure 6), most commonly involving GCS. Combination regimens with two agents were somewhat less frequent, while the use of three or more drugs was comparatively rare.

The analysis showed no significant changes in the frequency of off-label use dynamics over the years (Cochran–Armitage Trend Test, *p* = 0.841).

## 4. Discussion

This study revealed several epidemiological patterns that are consistent with previously reported findings, reinforcing the reliability of our cohort characteristics and their relevance to clinical practice. A high prevalence of dermatological comorbidities, particularly AD and, to a lesser extent, psoriasis, was observed, corresponding with results from earlier studies that have identified an association between AA and other immune-mediated skin conditions [18,19]. Interestingly, women constituted the majority of the study population and were, on average, older than male patients (mean age 27 vs. 19.2 years, *p* = 0.01), although both groups remained under the age of 30. While the first two observations differ from existing literature—which generally reports an equal prevalence of AA between sexes—the latter finding is consistent with previously published data [4,19]. Although sex-related differences were noted, the underlying factors remain unclear, as potential biological and psychosocial contributors were not examined in this study. While the treatment type or gender distribution did not vary significantly across residence categories, the urban predominance in patient residence (72.9%, *p* < 0.001) was observed.

A notable aspect observed in this study is the predominance of off-label therapy among patients with AA. Except for a single patient, all individuals receiving pharmacological treatment were managed with off-label drugs. This finding underscores the high prevalence of off-label medication use in clinical practice, particularly within the European context. A likely explanation for this trend might be the limited range of licensed therapies. Throughout the study period (2014–2024), only two medications were registered for the treatment of AA—baricitinib and ritlecitinib. Notably, both approvals occurred in 2024, near the end of the observation period. Interestingly, no significant changes were observed over time in patient numbers, gender distribution, or the use of off-label therapies—even following the introduction of newly authorized treatments. It could indicate persistent barriers to adopting newer drugs, particularly related to their high cost. In Poland no funding for therapies in accordance with the SmPCs was available during the analyzed period. Reimbursement for one of the on-label therapies, ritlecitinib, commenced in July 2025—after the study period concluded [20]. Importantly, even with the introduction of funding, a substantial proportion of patients may continue to receive off-label treatments. This is due to strict inclusion criteria, which require documented failure or intolerance of at least one prior systemic therapy before initiating the reimbursed on-label option [20]. As such, patients are required to undergo off-label treatment before qualifying for funded on-label therapy. This issue is not exclusive to Poland. At the time of Poland’s reimbursement application in 2024, only one other EU country provided funding for AA treatment, and only under the condition of prior systemic corticosteroid use, either as monotherapy or in combination with immunosuppressive agents [21]. These observations point to broader systemic challenges in the financing and accessibility of evidence-based AA therapies within the EU. Furthermore, the widespread use of off-label treatments raises some concerns, particularly in relation to occurrence of adverse events. Given the lack of regulatory oversight for such therapies in this indication, it may be prudent to implement formal monitoring mechanisms to track and manage potential complications arising from off-label pharmacotherapy in AA.

The proportion of patients who did not receive therapeutic intervention, accounting for 21.6% of the AA cohort, is also described. This subgroup predominantly consisted of individuals who presented for diagnostic assessment only and did not require or pursue further management. In most cases, these patients attended a single outpatient visit, typically for confirmation of the diagnosis, counseling, or evaluation of disease severity, without subsequent initiation of treatment or follow-up.

Analysis of the specific drug classes revealed a heterogeneous treatment landscape, highlighting the varied therapeutic strategies adopted in managing AA. Due to the absence of clinical outcome measures in this study, it is not possible to assess treatment effectiveness or safety; therefore, the following section provides a brief literature-based overview of the drug classes observed in the analyzed alopecia areata treatment regimens along with the frequency of their use.

GCS were the most used drugs, forming part of the therapeutic regimen in 65.3% of cases. Various formulations, including topical steroids, intralesional injections, and systemic corticosteroids are reported in the literature for managing AA [2,4,22,23,24]. However, their use is not without side effects, as they may lead to skin atrophy, folliculitis, and adrenal suppression for topical use, and hypertension, weight gain, and Cushingoid features for systemic administration [2,4,24]. TCIs represented another group of medications, used in the 17.6% of therapy regimens. Their role in AA treatment remains controversial, as current literature suggests limited efficacy in promoting hair regrowth in alopecic patches [2,23,25,26]. Prostaglandin analogs were used in 11.1% of cases. Studies report their potential to stimulate hair regrowth on the scalp and eyelashes, although localized side effects, particularly near the eyes, may occur [2,27]. Immunosuppressive drugs, including methotrexate and cyclosporine, were also employed (9.7% of therapy regimens). Supporting evidence for their efficacy in AA remains limited, yet the available literature mostly suggests the effectiveness of these agents, while recognizing their adverse effects, including increased infections risk, gastrointestinal upset, nephrotoxicity and hematologic deviations [2,4,23,28,29,30].

In the analyzed cohort, other pharmacological treatments for AA were used in 39.4% of cases. Among these, minoxidil was the most frequently applied agent in the examined population, generally reported in the literature as adjunctive rather than monotherapy [31,32]. Different drugs categorized as “Other” applied in the studied population, such as mesalazine, hydroxychloroquine and various contact immunotherapies, have been less extensively investigated, and the available evidence regarding their effectiveness remains scarce and/or inconclusive [23,32,33,34,35,36]. The widespread use of these therapeutic options likely reflects the limited availability of established, effective treatments, which may contribute to the adoption of less evidence-based approaches.

Janus kinase inhibitors were among the least frequently used medications in the study group, being included in 4.2% of therapies, with 2.8% being baricitinib or ritlecitinib. This confirms previous considerations: although registered medications exist, their availability remains low, indicating ongoing challenges in implementing them in clinical practice. Therefore, even though the potential registration of new drugs—such as deuruxolitinib, currently approved only by the FDA—offers hope for expanding the range of therapeutic options for AA, it is important to emphasize that such approvals must be accompanied by systemic changes in drug financing and patient eligibility for treatment [37].

This research is subject to several limitations. Its retrospective design and reliance on EMR may have led to incomplete data, as paper-based records were excluded. Additionally, the analysis only considered off-label use based on indication mismatches with the SmPC, potentially overlooking cases related to age, dosage, pharmaceutical form, or route of administration. This may have resulted in a misjudgment of frequency of the outside the registration use of drugs. The study also lacked data on treatment outcomes, disease severity, patient adherence, and satisfaction, limiting the ability to assess the broader clinical impact of the therapies.

## 5. Conclusions

This study offers a detailed insight into the epidemiological and therapeutic landscape of alopecia areata in a real-world European setting. Our findings demonstrate a marked predominance of off-label pharmacotherapy, reflecting both the historical lack of approved treatments and the persistent barriers to the adoption of newly authorized options. These results underscore the need for systemic efforts aimed at improving access to licensed medications, ensuring equitable care, and establishing structured monitoring of off-label drug use to enhance patient safety and therapeutic outcomes in alopecia areata.

## Figures and Tables

**Table 1:** Drugs used in the treatment of alopecia areata.

Group of Medications	Drug, Concentration (%)	SmPC Indication for AA Treatment
GCS *	Hydrocortisone 1%, Prednisolone 0.5%, Flumetasone pivalate 0.02%, Triamcinolone acetonide 0.1%, Hydrocortisone butyrate 0.1%, Fluticasone propionate 0.05%, Alclometasone 0.05%, Fluocinolone acetonide 0.025%, Methylprednisolone aceponate 0.1%, Mometasone furoate 0.1%, Betamethasone dipropionate 0.05%, Clobetasol propionate 0.05%, Methylprednisolone, Prednisone, Prednisolone, Dexamethazone	no
TCI **	Tacrolimus 0.1%, Tacrolimus 0.03%, Pimecrolimus	no
Others	Apremilast, Botulinum toxin, Contact immunotherapies (Diphenylcyclopropenone, Squaric acid dibutyl ester), Imiquimod, Hydroxychloroquine, Chloroquine, Minoxidil, Sulfasalazine, Mesalazine, Sildenafil, Topical nitrogen mustard	no
Immunosuppressants	Methotrexate, Cyclosporine	no
Prostaglandins	Bimatoprost, Latanoprost	no
JAK inhibitors	Tofacitinib, Ruxolitinib, Upadacitinib	no
JAK inhibitors	Baricitinib, Ritlecitinib	yes

* GCS—glucocorticosteroids; ** TCI—topical calcineurin inhibitors.

## Data Availability

The original contributions presented in this study are included in the article. Further inquiries can be directed to the corresponding authors.

## Abbreviations

The following abbreviations are used in this manuscript:

AA	Alopecia Areata
AAS	Alopecia Areata Scale
AD	Atopic Dermatitis
CMH	Cochran–Mantel–Haenszel
DLQI	Dermatology Life Quality Index
EMA	European Medicines Agency
EMR	Electronic Medical Records
EU	European Union
FDA	Food and Drug Administration
JAK	Janus Kinase
Ps	Psoriasis
PA	Psoriatic Arthritis
SALT	Severity of Alopecia Tool
SmPC	Summary of Product Characteristic
